# Optogenetic clustering of CNK1 reveals mechanistic insights in RAF and AKT signalling controlling cell fate decisions

**DOI:** 10.1038/srep38155

**Published:** 2016-11-30

**Authors:** Adrian Fischer, Bettina Warscheid, Wilfried Weber, Gerald Radziwill

**Affiliations:** 1 Department of Biochemistry and Synthetic Biology, Faculty of Biology, University of Freiburg, Schänzlestr. 18, 79104 Freiburg, Germany; 2 Department of Biochemistry and Functional Proteomics, Faculty of Biology, University of Freiburg, Schänzlestr. 1, 79104 Freiburg, Germany; 3 BIOSS - Centre for Biological Signalling Studies, University of Freiburg, Schänzlestr. 1, 79104 Freiburg, Germany

## Abstract

Scaffold proteins such as the multidomain protein CNK1 orchestrate the signalling network by integrating and controlling the underlying pathways. Using an optogenetic approach to stimulate CNK1 uncoupled from upstream effectors, we identified selective clusters of CNK1 that either stimulate RAF-MEK-ERK or AKT signalling depending on the light intensity applied. OptoCNK1 implemented in MCF7 cells induces differentiation at low light intensity stimulating ERK activity whereas stimulation of AKT signalling by higher light intensity promotes cell proliferation. CNK1 clustering in response to increasing EGF concentrations revealed that CNK1 binds to RAF correlating with ERK activation at low EGF dose. At higher EGF dose active AKT binds to CNK1 and phosphorylates and inhibits RAF. Knockdown of CNK1 protects CNK1 from this AKT/RAF crosstalk. In C2 skeletal muscle cells CNK1 expression is induced with the onset of differentiation. Hence, AKT-bound CNK1 counteracts ERK stimulation in differentiated but not in proliferating cells. Ectopically expressed CNK1 facilitates C2 cell differentiation and knockdown of CNK1 impaired the transcriptional network underlying C2 cell differentiation. Thus, CNK1 expression, CNK1 clustering and the thereto related differential signalling processes decide on proliferation and differentiation in a cell type- and cell stage-dependent manner by orchestrating AKT and RAF signalling.

Cells process numerous signals, originating from internal biological events or the environment to generate the appropriate cellular response. Signal transduction networks relay information by pathways that are highly interconnected with each other. Positive and negative feedback mechanisms as well as crosstalks control the signal output and decide on the cell fate and cellular behaviour. Scaffold proteins comprising multiple protein-protein interaction domains act as signalling hubs recruiting upstream and downstream elements and thereby integrate and mediate information^1^.

The scaffold proteins of the connector enhancer of KSR (CNK) family are multidomain proteins without an enzymatic function and conserved from invertebrates to vertebrates (Fig. 1A)^2^,^3^. The N-terminus consists of the three protein-protein interaction domains: a sterile alpha motif (SAM), a conserved region of CNK (CRIC) and a post synaptic density protein/Drosophila disc large tumour suppressor/zonula occludens-1 protein (PDZ). The C-terminus harbours a pleckstrin homology (PH) region and a coiled-coil (CC) domain. While invertebrates express only one isoform, vertebrates express three CNK isoforms. CNK1 is ubiquitously expressed, CNK2 is mainly found in neuronal cells, and CNK3 is not well characterized so far. CNK1 is the best studied CNK family member coordinating signal transmission of several signal pathways depending on the stimulus and cell type^3^. CNK1 binds to the GTPase RHO and mediates RHO-dependent stimulation of the Jun N-terminal kinase (JNK)^4^,^5^. CNK1 interacts with RAF in growth factor-stimulated and oncogenic-activated cells and mediates SRC-dependent activation of CRAF in vascular endothelial growth factor (VEGF)-stimulated cells^6^. CNK1 drives AKT-dependent cell proliferation and co-localizes with AKT at the plasma membrane in invasive breast cancer tumours^7^. In addition, CNK1 promotes invasion of cancer cells by AKT-dependent NFκB pathway activation^8^. Insulin recruits CNK1 complexed with ARF guanine nucleotide exchange factors of the cytohesin family to the plasma membrane facilitating PI3K/AKT signalling^9^. In PDGF stimulated cells, differential tyrosine phosphorylation of CNK1 controls the oligomerization state of CNK1 and its subcellular localization as well as CNK1-induced cell proliferation and gene expression^10^.

Optogenetics provides tools to stimulate signalling by oligomerization and membrane-recruitment of signalling proteins or reconstitution of split proteins in a light-dependent manner^11^,^12^. Previous work indicates that oligomerization induced by growth factors and activating mutants affects CNK1 signalling^6^,^10^. We chose an optogenetic approach to precisely control the oligomerization state of CNK1 to study CNK1-mediated signalling uncoupled from upstream signalling induced in a time-resolved manner. The optogenetic approach used in this study is based on the reversible homooligomerisation of the photolyase homology region (PHR, amino acids 1-498) of the *Arabidopsis thaliana* photoreceptor cryptochrome 2 (CRY2). PHR-CRY2 (abbreviated hereafter as CRY2) oligomerises within seconds upon exposure to blue light of 460 nm wavelength and dissociates within minutes in the dark^13^,^14^,^15^. This approach has been successfully used to induce signalling by CRY2-mediated oligomerization of chimeric RAF proteins or chimeric fibroblast growth factor receptors (FGFR)^16^,^17^,^18^ and by indirect oligomerization of endogenous receptor tyrosine kinases including FGFR, platelet-derived growth factor receptor (PDGFR) or integrins^19^. Using light-controllable CNK1, optoCNK1, we could demonstrate that dependent on the light intensity applied CNK1 acts as platform for different signalling complexes and allows switching between stimulation of ERK and AKT signalling. Furthermore, we show that similar to the light intensity the dose of epidermal growth factor induces a change in CNK1 complex composition and thereby allows RAF/ERK signalling or exertion of an AKT/RAF crosstalk which suppresses RAF/ERK signalling. Analysing C2 skeletal muscle cells and MCF7 breast cancer cells we demonstrate that CNK1 expression and CNK1-mediated signalling decides on proliferation *versus* differentiation in a cell type- and cell stage-dependent manner.

## Results

### Light-activatable CNK1 specifically stimulates RAF/ERK and AKT signalling

**Figure 1:**
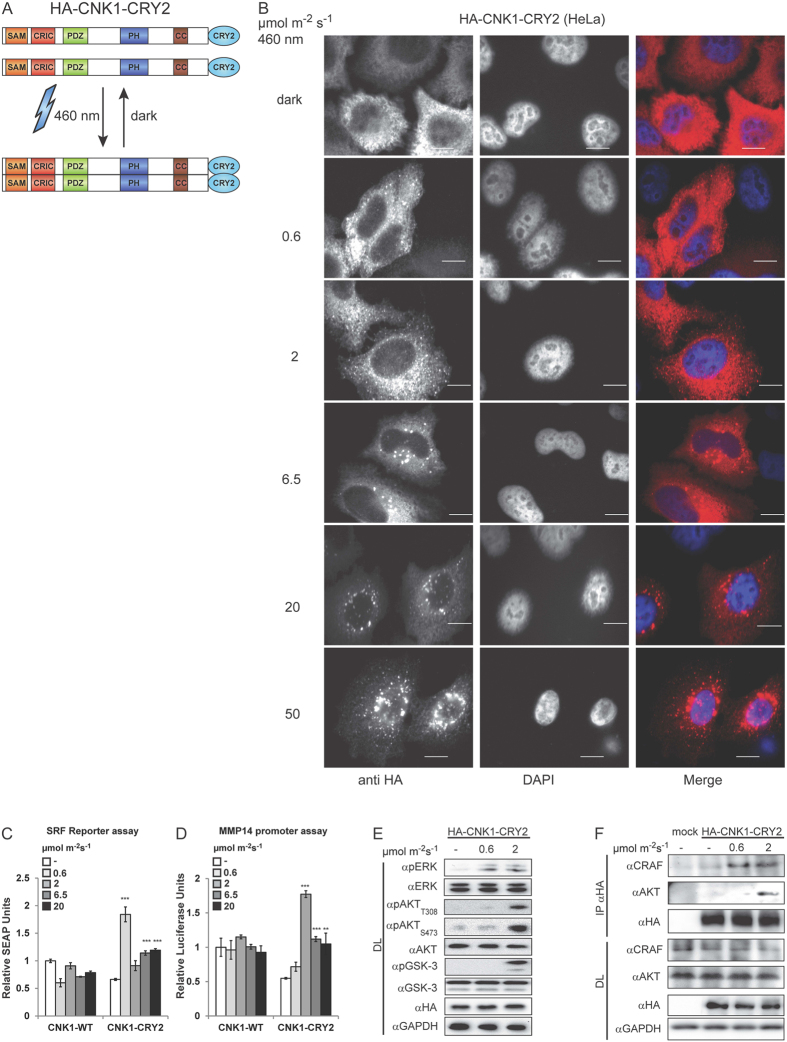
Clustering of CNK1-CRY2 stimulates RAF/ERK and AKT signalling. (**A**) Scheme of light-controlled oligomerization of CNK1-CRY2. (**B**) Immunostaining shows increased clustering of HA-CNK1-CRY2 with increased light intensity at 460 nm. Left: anti-HA antibody for HA-CNK1-CRY2, middle: DAPI for nuclear staining, right: merge images, scale bar: 10 μm. (**C**) HA-CNK1-CRY2 expressing HEK293T cells preferentially activates SRF-dependent reporter upon illumination with 460 nm blue light activity at 0.6 μmol m^−2^ s^−1^. N = 3, mean + SEM, two-tailed Students *t-test*, ***p < 0.001. See Supplementary Figure S8 for control of protein expression. (**D**) HA-CNK1-CRY2 expressing HEK293T cells preferentially activate MMP14 promoter-dependent reporter gene expression upon illumination with 460 nm blue light activity at 2 μmol m^−2^ s^−1^. N = 3, mean + SEM, two-tailed Students *t-test*, **p < 0.01 ***p < 0.001. See Supplementary Figure S8 for control of protein expression. (**E**) HA-CNK1-CRY2 activated with 0.6 μmol m^−2^s^−1^ for 15 min increased the level of phosphorylated ERK1/2 (pERK) whereas activation with 2 μmol m^−2^ s^−1^ additionally increased phosphorylation of AKT (pAKT_T308_) and of the AKT substrate GSK-3 (pGSK-3_S21_). (**F**) Co-precipitation experiments show that CRAF co-precipitates with HA-CNK1-CRY2 upon illumination with 0.6 μmol m^−2^ s^−1^ for 15 min and additionally with AKT upon illumination with 2 μmol m^−2^ s^−1^; see also Supplementary Figure S2.

Stimulation of cells with growth factors or co-expression of oncogenic RAS_G12V_ triggers oligomerization of CNK1^6^,^10^. To study the biological impact of oligomeric CNK1 uncoupled from upstream signals, we generated optoCNK1 based on CNK1 fused to PHR-CRY2 (CNK1-CRY2) (Fig. 1A). CNK1-CRY2 expressed in HeLa cells clusters upon irradiation with blue light (Fig. 1B). The cluster size of CNK1-CRY2 increased with the light intensity applied and irradiation with blue light of 0.6 μmol m^−2^ s^−1^ for 15 min already induced clusters of chimeric CNK1 detectable by immunostaining (Fig. 1B). It should be mentioned that the highest light dose tested, 50 μmol m^−2^ s^−1^, impaired the cells and lowered their viability upon longer irradiation.

To get a first hint whether oligomerization and the cluster size affects CNK1-mediated signalling, we performed pathway-specific reporter assays. Assays with secreted embryonic alkaline phosphatase (SEAP) as reporter under the control of the serum response factor (SRF) activated by ERK signalling were performed to monitor the RAF-MEK-ERK pathway^20^. Matrix metalloproteinase 14 (MMP14) promoter-controlled luciferase reporter assays were used to measure AKT signalling^8^. Inhibitor treatment confirmed that SRF reporter activation depends on ERK signalling and MMP14 promoter activation on AKT signalling (Supplementary Figure S1A,B). The lowest light dose tested, 0.6 μmol m^−2^ s^−1^, led to the highest increase of the SEAP reporter activity (three-fold) compared to the dark control (Fig. 1C; Supplementary Figure S8, see related to Fig. 1). Higher light intensities, up to 20 μmol m^−2^ s^−1^, resulted in a less than two-fold increase compared to the dark control. In contrast, irradiation with 0.6 μmol m^−2^ s^−1^ only marginally induced the MMP14 promoter activity, whereas irradiation with 2 μmol m^−2^ s^−1^ induced a 3.5-fold increased reporter activation compared to the dark control (Fig. 1D; Supplementary Figure S8, see related to Fig. 1). Increasing light intensity diminished the MMP14 promoter-dependent reporter activity again. Thus, CNK1-CRY2 activated by 0.6 μmol m^−2^ s^−1^ preferentially stimulates ERK signalling and CNK1-CRY2 activated by 2 μmol m^−2^ s^−1^ preferentially AKT signalling. Consistently, CNK1 elevated phosphorylation of AKT at its activation sites Thr308 and Ser473 and of the AKT substrate GSK-3 α/β in cells exposed to 2 μmol m^−2^ s^−1^ for 15 min (Fig. 1E). Phosphorylation of ERK was already detectable at a light intensity of 0.6 μmol m^−2^ s^−1^ and remained unchanged at 2 μmol m^−2^ s^−1^ (Fig. 1E). In addition, exposure of cells with 0.6 μmol m^−2^ s^−1^ for 15 min induced binding of CRAF, but not of AKT to CNK1-CRY2, whereas exposure to 2 μmol m^−2^ s^−1^ enabled interaction of CNK1-CRY2 with AKT and CRAF (Fig. 1F). Accompanied with AKT we detected PDK1, the kinase phosphorylating AKT at Thr308, in the CNK1 complex (Supplementary Figure S2). SIN1, a key component of mTORC2 that is activated by AKT and involved in a positive feedback loop phosphorylating AKT at Ser473^21^, could not be detected under the conditions tested (Supplementary Figure S2). Taken together, the precise light-dependent control of optoCNK1 clustering correlates with changes in complex formation and can be used to switch between ERK and AKT signalling.

### Kinetics of optoCNK1-mediated ERK and AKT activation

**Figure 2:**
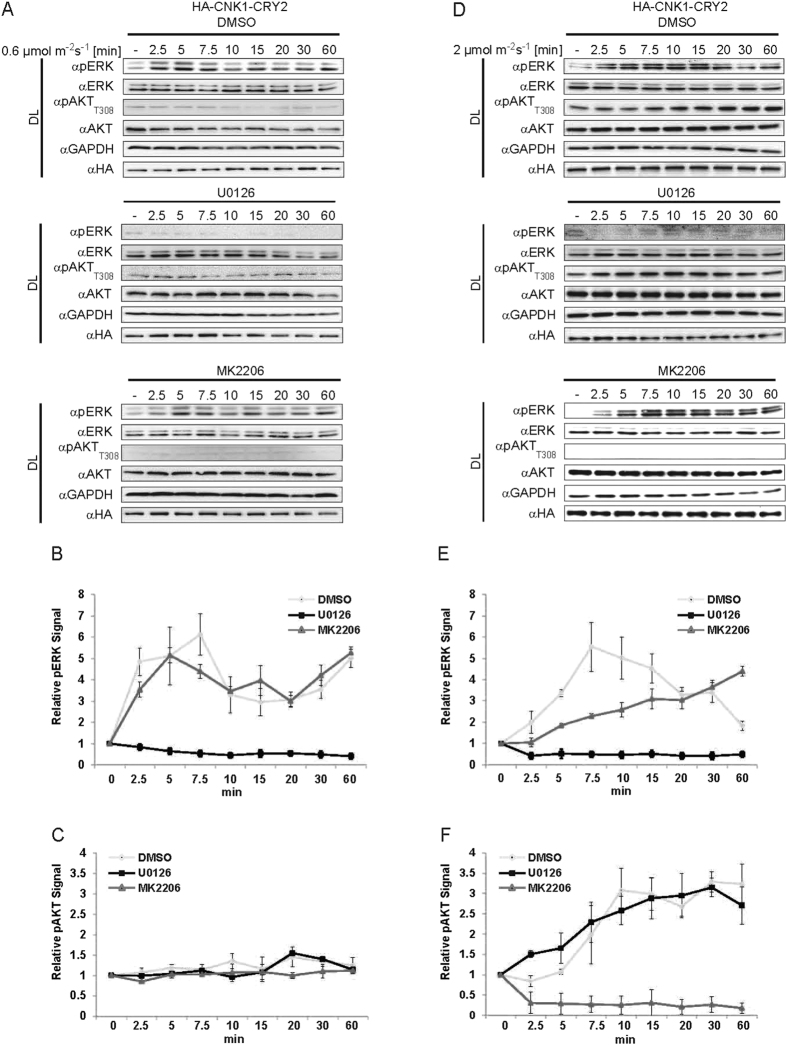
Kinetics of CNK1-CRY2 mediated ERK and AKT activation. (**A** and **D**) HA-CNK1-CRY2 expressing HEK293T cells were treated with DMSO (upper panels), U0126 (middle panels) or MK2206 (lower panel) and illuminated with 0.6 *μ*E m^−2^ s^−1^ of 460 nm (**A**) and 2 *μ*E m^−2^ s^−1^ (**D**) for the time points indicated. Immunoblots show that the kinetics of ERK and AKT phosphorylation depend on the light intensity used for activation of HA-CNK1-CRY2. (**B,C**) and (**E,F**) Quantification of immunoblot data shown in (**A**) and (**D**). N = 3, mean + SEM.

Next, we analysed optoCNK1-mediated ERK and AKT activation induced by low or high light intensities in a time-resolved manner. To this end, we pre-treated HA-CNK1-CRY2 expressing HEK293T cells with the MEK inhibitor U0126, the AKT inhibitor MK2206 or the solvent DMSO before irradiation with 0.6 or 2 μmol m^−2^ s^−1^ for 2.5 min up to 60 min. Irradiation of cells with low light intensity first led to activation of ERK reaching a maximum at 5 to 7.5 min (Fig. 2A,B). Within the next 20 min, the pERK level declined but afterwards increased again to the level of the first peak. This course of the pERK level was not affected by the AKT inhibitor MK2206 (Fig. 2A,B). Treatment of cells with the MEK inhibitor U0126 prevented ERK phosphorylation as expected. Regarding AKT stimulation, low light intensity did not elevate the level of pAKT_T308_ within the first 15 min (Fig. 2A and C). After 15 min the pAKT_T308_ level slightly increased and declined again within the next 40 min. Treatment with MK2206 prevented AKT phosphorylation, whereas U0126 had no effect (Fig. 2A and C). Irradiation with 2 μmol m^−2^ s^−1^ led to a constant increase in the pAKT_T308_ level up to 10 min before reaching a plateau (Fig. 2D and F). AKT activation was not influenced by U0126 and effectively inhibited by MK2206 (Fig. 2D and F). The pERK level increased within the first 7.5 min upon irradiation with 2 μmol m^−2^ s^−1^ and slowly returned to the basal level within the next 50 min (Fig. 2D,E). U0126 prevented ERK activation. Interestingly, treatment with MK2206 gradually increased the pERK level during the 60 min tested (Fig. 2D,E). Thus, inhibition of AKT, activated by optoCNK1 at higher light intensity, seems to restore ERK activity. This indicates that CNK1 mediates a functional interaction between the AKT and the ERK pathway that results in AKT-dependent inhibition of RAF signalling.

### The SAM domain of CNK1 is essential to mediate AKT but not ERK signalling

**Figure 3:**
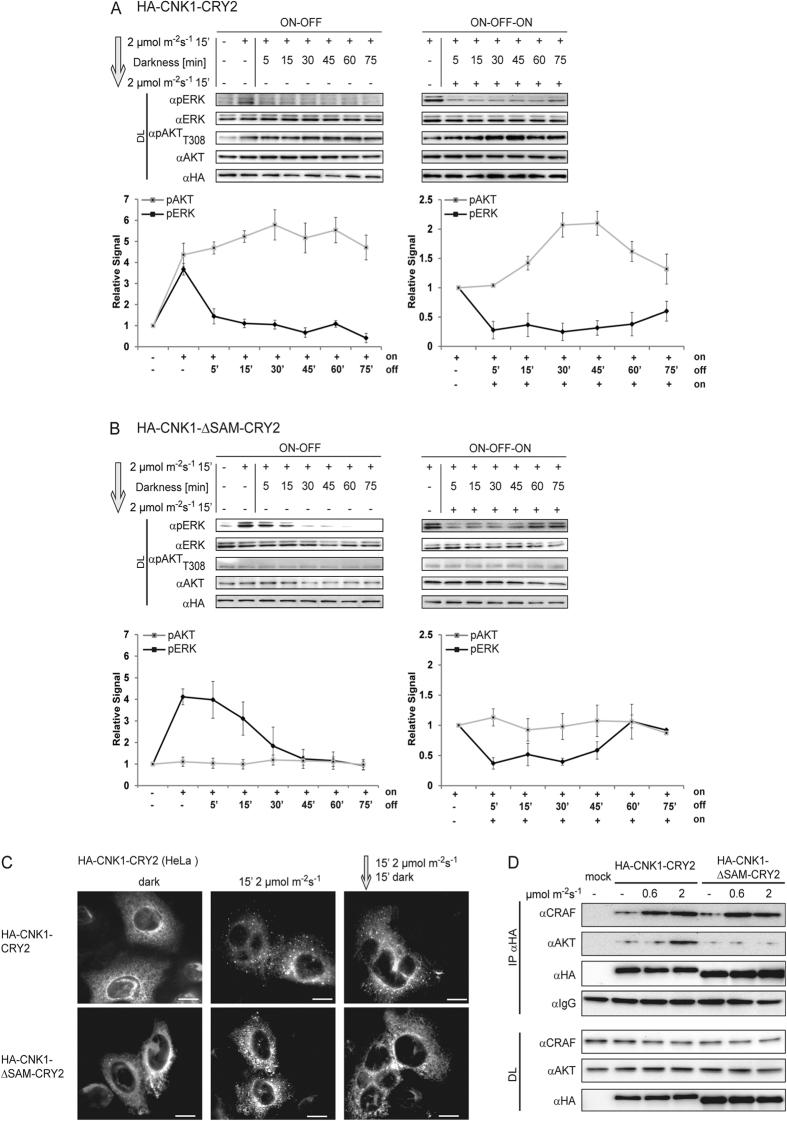
The SAM domain of CNK1-CRY2 is essential for mediating the AKT/RAF crosstalk. (**A**) ON-OFF (left) and ON-OFF-ON (right) kinetics of HA-CNK1-CRY2 activated by illumination with 460 nm and 2 *μ*E m^−2^ s^−1^ for the time points indicated monitored by ERK and AKT phosphorylation. Charts represent quantification of pERK and pAKT immunoblot data of three independent experiments. (**B**) ON-OFF (left) and ON-OFF-ON (right) kinetics of HA-CNK1_ΔSAM_-CRY2 activated by illumination with 460 nm and 2 *μ*E m^−2^ s^−1^ for the time points indicated monitored by ERK and AKT phosphorylation. Charts represent quantification of pERK and pAKT immunoblot data of three independent experiments. (**C**) Immunostaining of HA-CNK1-CRY2 and HA-CNK1_ΔSAM_-CRY2 overexpressed in HeLa cells demonstrated that the presence of the SAM domain delayed dissociation of CNK1-CRY2 clusters induced by illumination with 460 nm and 2 *μ*E m^−2^ s^−1^ for 15 min. Left: anti-HA antibody for HA-CNK1-CRY2 and HA-CNK1_ΔSAM_-CRY2, middle: DAPI for nuclear staining, right: merge images (Red: anti-HA antibody; Blue: DAPI), scale bar: 10 μm. (**D**) HEK293T cells expressing HA-CNK1-CRY2 and HA-CNK1_ΔSAM_-CRY2 were light-stimulated as indicated. Deletion of the SAM domain abolished binding of AKT to CNK1 but did not affect binding of CRAF to CNK1.

To further analyse the dynamics of the CNK1-mediated stimulation of AKT and RAF/ERK signalling, we performed ON-OFF-ON kinetics. First, optoCNK1 expressing cells were irradiated with blue light at an intensity of 2 μmol m^−2^ s^−1^ for 15 min, followed by further incubation in the dark for different time points. The level of pAKT_T308_ obtained by 15 min irradiation remained stable during a period of 75 min in the dark (Fig. 3A, left). In contrast, the level of pERK decreased immediately under dark conditions. Next, we restimulated the cells incubated in the dark by a second exposure to light of the same intensity for 15 min. The second light pulse led to a significant increase of the pAKT_T308_ level best detectable after a dark phase of 30 to 45 min, whereas pERK remained at a low level (Fig. 3A, right). Our data demonstrate that optoCNK1 promotes AKT stimulation upon irradiation with 2 μmol m^−2^ s^−1^ correlating with reduced ERK activation.

CNK proteins contain a SAM domain at the N-terminus that heterodimerises with the SAM domain protein Hyphen/Aveugle^22^,^23^. In addition, the SAM domain of CNK1 is a target of PDGF-induced phosphorylation facilitating the clustering of CNK1 and CNK1-induced signalling^10^. In order to gain insights into the role of SAM domain in CNK1 signalling, we generated a light-activatableCNK1 lacking the SAM-domain, optoCNK1_ΔSAM_. While irradiation of optoCNK1_ΔSAM_ with 2 μmol m^−2^ s^−1^ strongly increased the level of pERK and delayed its decline, it did not induce AKT phosphorylation (Fig. 3B, left). A second light pulse enabled reactivation of ERK after a lag phase of 45 min in the dark. (Fig. 3B, right). This emphasizes the importance of CNK1, and especially its SAM domain, in mediating the inhibitory effects of AKT on ERK signalling. Prolonged AKT activation depends on the presence of the SAM domain and light-induced clusters of optoCNK1 remained stable in the dark (Fig. 3C). OptoCNK1_ΔSAM_ clustered upon irradiation and dissociated in the dark within 15 min typical for CRY2-mediated clustering (Fig. 3C). Thus, the SAM domain antagonizes the reversibility of CRY2 clustering in the dark and prolonged cluster formation of the CNK1-CRY2 chimeric protein. Consistently, we found that the SAM domain is crucial for binding of AKT to light-induced CNK1 oligomers. Under conditions CNK1-CRY2 interacted with AKT, CNK1_ΔSAM_-CRY2 lost its ability of AKT binding (Fig. 3D).

### OptoCNK1 decides on cell fate decision in MCF7 cells

**Figure 4:**
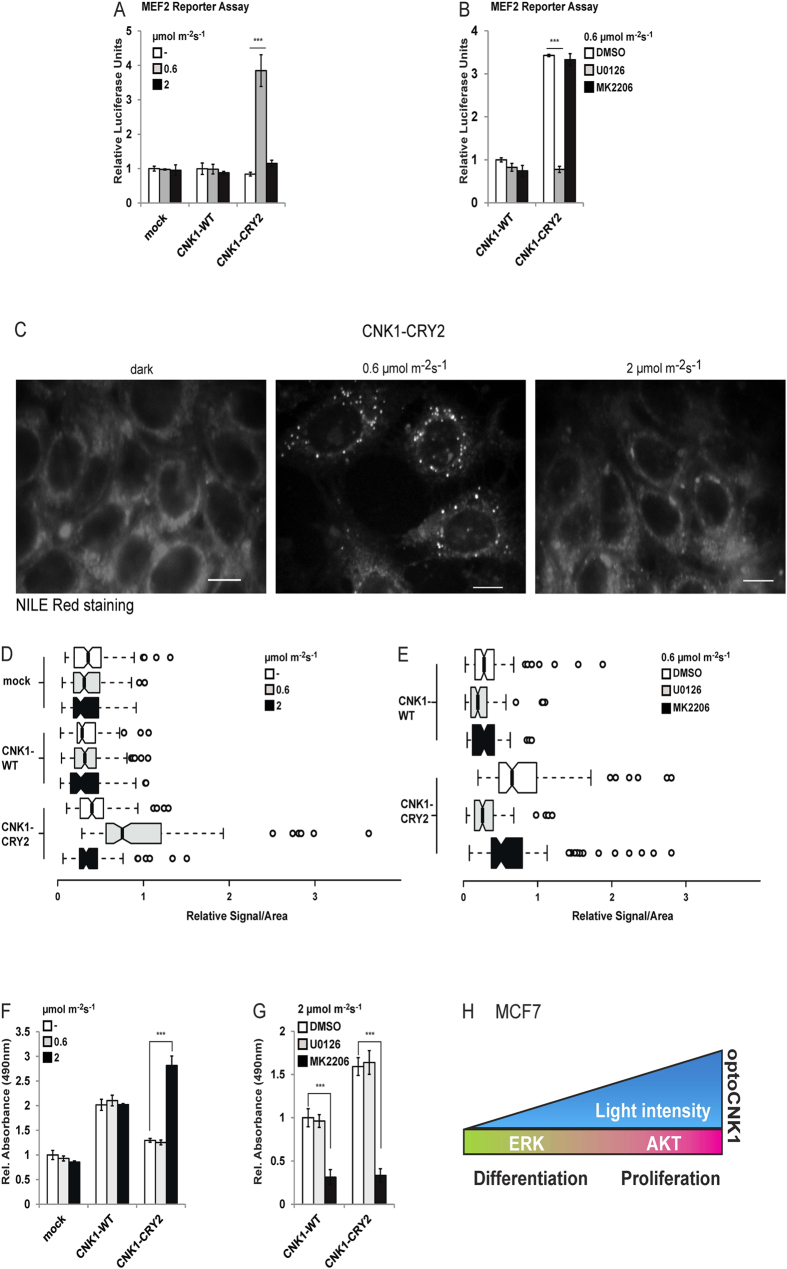
Activation level of OptoCNK1 decides on cell proliferation and differentiation of MCF7 cells. (**A**) CNK1-CRY2 expressed in MCF7 cells stimulates MEF2-dependent luciferase reporter gene expression upon exposure with light of 460 nm and 0.6 μmol m^−2^ s^−1^ for 24 h. N = 3, mean + SEM, two-tailed Students *t-test*, ***p < 0.001. See Supplementary Figure S8 for control of protein expression. (**B**) The MEK inhibitor U0126 but not the AKT inhibitor MK2206 prevents CNK1-CRY2-induced MEF2 reporter gene expression. N = 3, mean  + SEM, two-tailed Students *t-test*, ***p < 0.001. See Supplementary Figure S8 for control of protein expression. (**C**) Stimulation of CNK1-CRY2 by illumination with 460 nm light and 0.6 μmol m^−2^ s^−1^ for 36 h induced differentiation of MCF7 cells monitored by Nile Red staining to detect lipid droplets. (**D**) Quantification of Nile Red stained MCF7 cells demonstrated that CNK1-CRY2 stimulated with 0.6 μmol m^−2^ s^−1^ for 24 h induced differentiation. N = 100 of four independent experiments each. (**E**) CNK1-CRY2-induced differentiation of MCF7 cells is abolished by the MEK inhibitor U0126. N = 100 of four independent experiments each. (**F**) CNK1-CRY2 activated by 2 μmol m^−2^ s^−1^ for 36 h promotes proliferation of MCF7 cells as monitored by an MTT assay. N = 3, mean + SEM, two-tailed Students *t-test*, ***p < 0.001. (**G**) The AKT inhibitor MK2206 but not the MEK inhibitor U0126 prevents MCF7 cell proliferation induced by CNK1-CRY2 activated by 2 μmol m^−2^ s^−1^ for 36 h. N = 3, mean + SEM, two-tailed Students *t-test*, ***p < 0.001. (**H**) Scheme showing that signal strength of OptoCNK1 decides on the cell fate of MCF7 cells.

In MCF7 breast adenocarcinoma cells, AKT signalling promotes proliferation and RAF/ERK signalling differentiation^24^. To study the impact of CNK1 signalling on the cell fate we implemented optoCNK1 in MCF7 cells. First, we analysed optoCNK1 for inducing myocyte enhancer factor-2 (MEF2)-dependent gene expression in MCF7 cells. MEF2 is a widely expressed transcription factor induced during cell differentiation that controls cell differentiation and organogenesis^25^. MCF7 cells co-transfected with the CNK1-CRY2 construct and the MEF2 reporter were irradiated with 0.6 μmol m^−2^ s^−1^ and 2 μmol m^−2^ s^−1^ for 24 h. Only optoCNK1 stimulated by the low light dose activated MEF2-dependent reporter activity (Fig. 4A; Supplementary Figure S8, see related to Fig. 4). The MEK inhibitor U0126, but not the AKT inhibitor MK2206 prevented this reporter activation (Fig. 4B; Supplementary Figure S8, see related to Fig. 4). This confirms that optoCNK1 exposed to low light intensity stimulated MEF2-dependent gene expression through ERK signalling. Differentiation of MCF7 cells can be monitored by Nile red fluorescence staining of lipid droplets generated during differentiation^26^,^27^,^28^. And indeed, irradiation of MCF7 cells expressing optoCNK1 with low light dose for 36 h induced formation of lipid droplets (Fig. 4C,D). In contrast, no lipid droplets were detectable upon exposure of cells to the higher light dose or in the dark control. Stimulation of optoCNK1 by exposure to 0.6 μmol m^−2^ s^−1^ in the presence of U0126 prevented differentiation of MCF7 cells, while MK2206 did not. (Fig. 4E). Stimulation of CNK1-CRY2 expressing cells by exposure to 2 μmol m^−2^ s^−1^ led to increased cell proliferation, an effect inhibited by MK2206 treatment (Fig. 4F,G). Thus, optoCNK1 stimulated by the low light dose induces ERK signalling accompanied with differentiation of MCF7 cells. Contrary, optoCNK1 stimulated by the higher light dose activates AKT-dependent cell proliferation (Fig. 4H).

### CNK1 mediates AKT-dependent inhibition of CRAF in EGF-stimulated cells

**Figure 5:**
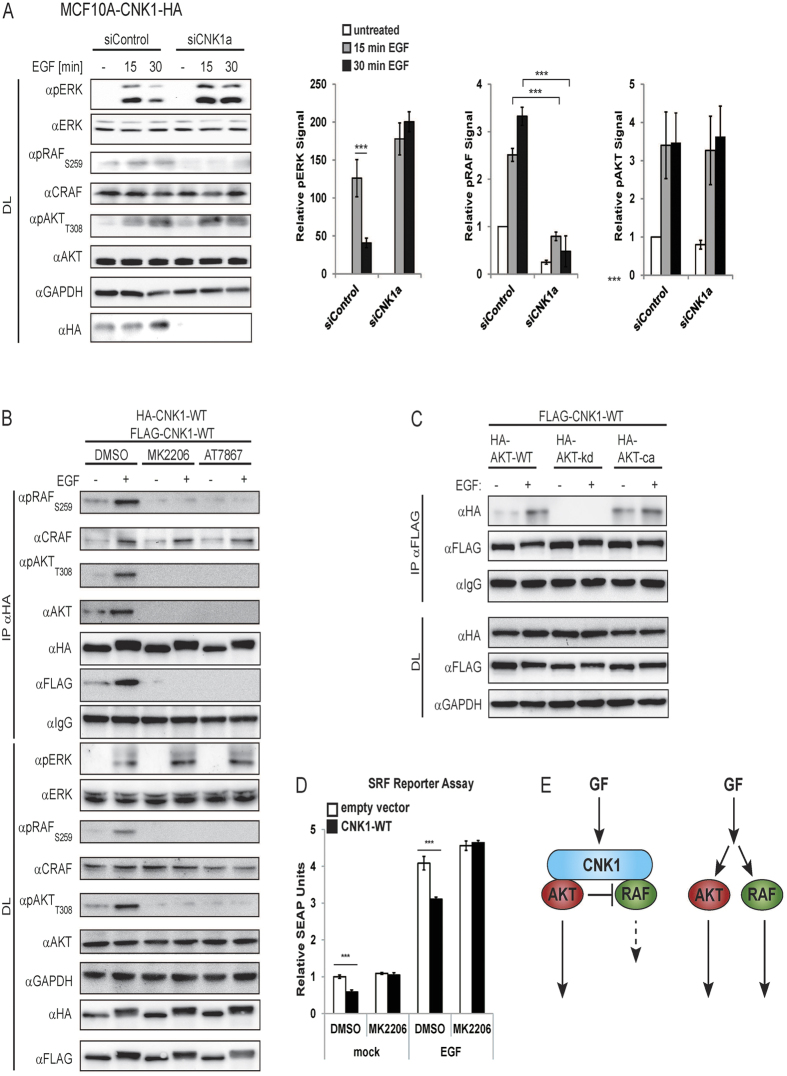
CNK1 mediates AKT-dependent inhibition of CRAF. (**A**) Knockdown of CNK1 in MCF10A-CNK1-HA cells abrogates AKT-dependent inhibitory phosphorylation of CRAF at Ser259 leading to increased ERK phosphorylation in EGF (20 ng/ml) stimulated cells. Bar charts represent quantified signals of three independent experiments. N = 3, mean + SEM, two-tailed Students *t-test*, ***p < 0.001. See also Supplementary Figure S3. (**B**) Treatment of HEK293T cells with the AKT inhibitors MK2206 or AT7867 abolished EGF (20 ng, 15 min)-induced binding of AKT and pT308AKT to CNK1 and also their residual interaction found in non-stimulated cells. (**C**) Wildtype AKT (HA-AKT-WT) and a constitutively active AKT mutant (HA-AKT-ca) bound to CNK1 in EGF-treated cells whereas a kinase-defective AKT mutant (HA-AKT-kd) failed to interact with CNK1. (**D**) Inhibition of AKT (MK2206) increased CNK1-induced SRF-dependent reporter gene expression (SEAP). N = 3, mean + SEM, two-tailed Students *t-test*, ***p < 0.001. See Supplementary Figure S8 for control of protein expression. (**E**) Scheme of the CNK1 mediated AKT/RAF crosstalk.

The kinetic studies with optoCNK1 revealed that CNK1 connects RAF-MEK-ERK and AKT signalling in a way that allows for inhibition of ERK activity by activated AKT (see Fig. 2). Previous studies have shown that AKT can inhibit CRAF activity by phosphorylation at Ser259^24^. To study whether CNK1 affects the AKT/RAF crosstalk leading to inhibition of CRAF by phosphorylation of Ser259, we knocked down CNK1 in MCF10A cells stably expressing HA-tagged CNK1 (Fig. 5A, siCNK1a; Supplementary Figure S3, siCNK1b). Cells were stimulated with epidermal growth factor (EGF) and analysed for the activation levels of AKT, CRAF and ERK by immunoblotting with the respective phospho-antibodies. In MCF10A-CNK1-HA cells treated with control siRNA (siControl) and stimulated with EGF, the level of pAKT_T308_ increased within 30 min (Fig. 5A, Supplementary Figure S3). According to elevated activation of AKT, CRAF phosphorylation at the inhibitory site Ser259 also increased. ERK showed a transient activation with a high phospho-ERK level at 15 min that significantly decreased during longer treatment with EGF (30 min). Thus, typical for the AKT/RAF crosstalk, initial AKT activity still allows RAF-dependent ERK activation, whereas prolonged AKT activity inhibits CRAF by phosphorylation of Ser259 accompanied with reduced ERK activity (see Fig. 5A)^29^. Knockdown of CNK1 by CNK1-specific siRNA impaired this AKT/RAF crosstalk. EGF-triggered activation of AKT did not lead to Ser259 phosphorylation and inhibition of CRAF and as consequence thereof inhibition of ERK. In contrast, EGF strongly stimulated ERK without reducing its activity upon longer treatment in CNK1 knockdown cells. Quantification of the results confirmed a 70% reduction of the pERK level in siControl cells treated for 30 min with EGF compared to cells stimulated for 15 min. Accordingly, the level of pCRAF_S259_ increased 30%. In cells stimulated with EGF for 30 min, knockdown of CNK1 led to an 80% decrease in the pCRAF_S259_ level, whereas the pERK level increased 5-fold compared to siControl cells (Fig. 5A, right panels). Knockdown of endogenous CNK1 in MCF10A parental cells and in HeLa cells consistently demonstrated that the depletion of CNK1 is sufficient to prevent AKT-mediated inhibition of CRAF and accompanied ERK inhibition (Supplementary Figure S3). Co-precipitation experiments confirmed that AKT and RAF interacted with CNK1 in EGF-treated cells (Fig. 5B). The presence of active pAKT_T308_ correlates with pRAF_S259_ in the CNK1 complexes. This indicates for a negative regulation of RAF by the AKT/RAF crosstalk. Inhibition of AKT activity by the allosteric inhibitor MK2206 preventing recruitment of AKT to the plasma membrane and the ATP competitive inhibitor AT7867 abolished the ability of AKT for CNK1 binding. CRAF binding to CNK1 was not affected by these AKT inhibitors. Consistent with the absence of AKT activity, CRAF was not phosphorylated at Ser259 (Fig. 5B). In agreement with the effects obtained by the AKT inhibitors, wildtype AKT and a constitutively active AKT mutant but not a kinase-defective AKT mutant were detected in EGF-induced CNK1 complexes (Fig. 5C). To functionally analyse the effect of ectopically expressed CNK1, we performed reporter assays with SEAP as reporter under the control of SRF that can be activated by ERK signalling^20^. Ectopic expression of CNK1 in HEK293T cells reduced SRF-dependent gene expression under basal conditions and in EGF-treated cells (Fig. 5D; Supplementary Figure S8, see related to Fig. 5). This CNK1-mediated inhibition of ERK signalling depends on AKT activity since treatment of cells with the AKT-specific inhibitor MK2206 completely restored ERK-dependent reporter gene expression in unstimulated as well as in EGF-treated cells. Taken together, these data demonstrate that CNK1 mediates the AKT/RAF crosstalk (Fig. 5E).

### EGF dose determines CNK1 complex formation and RAF/AKT switch in CNK1 signalling

**Figure 6:**
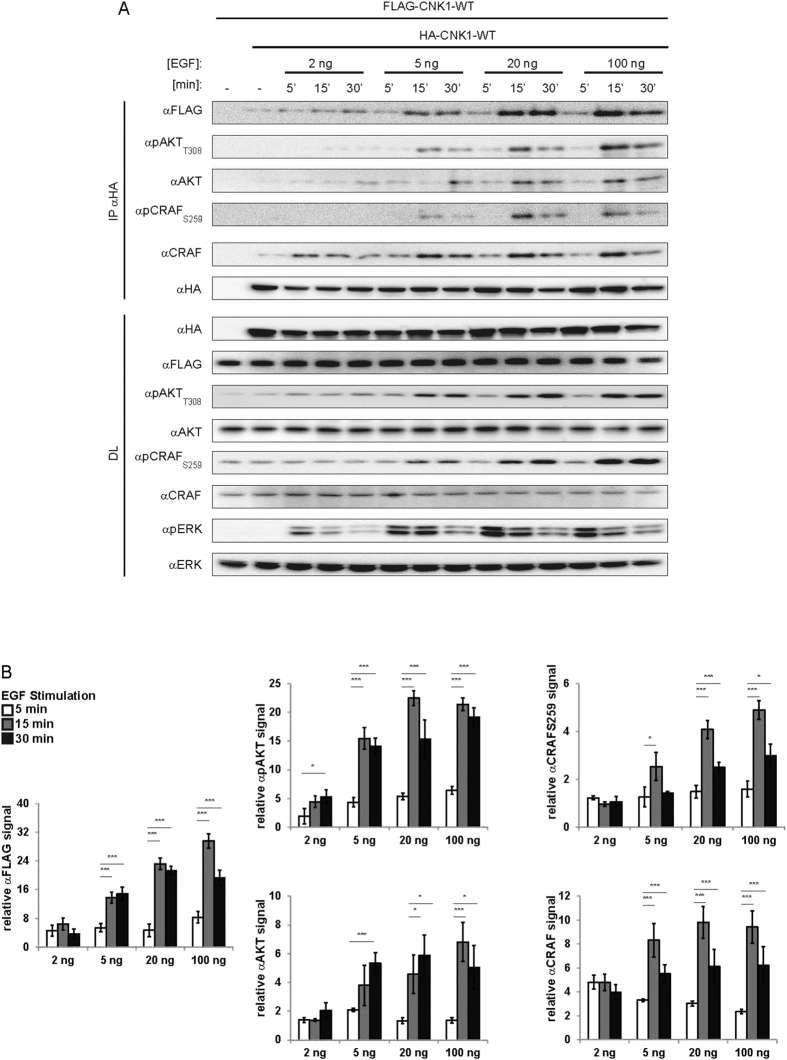
EGF dose determines CNK1 complex formation and RAF/AKT switch in CNK1 signalling. (**A**) HEK293T cells expressing FLAG-CNK1-WT and HA-CNK1-WT were treated with different doses of EGF for the time points indicated. Immunopurified HA-CNK1-WT (IP αHA) was analysed for co-precipitating FLAG-CNK1-WT and AKT and RAF proteins as indicated. Direct lysates (DL) were immunoblotted with the appropriate antibodies shown. (**B**) Quantification of co-precipitated proteins bound to HA-CNK1-WT. N = 3, mean + SEM, two-tailed Students *t-test*, *p < 0.05, ***p < 0.001. See Supplementary Figure S4 for quantification of direct lysates.

Complex composition and signalling of light-activatable CNK1 depends on the light intensity applied correlating with the cluster size of CNK1-CRY2 (see Fig. 1). The question arises whether the amount of growth factor used to stimulate cells affect CNK1 complex formation. To this end, HEK293T cells expressing HA-tagged and FLAG-tagged CNK1 were treated with an increasing amount of EGF for different time periods. First, we observed a slow rise of basal levels of HA-/FLAG-CNK1 complexes within the first 5 min followed by pronounced CNK1 co-precipitation within 15 min. Increased CNK1 oligomerization correlated with increased EGF doses (Fig. 6A, see Fig. 6B for quantification). In cells treated with 2 ng EGF for up to 30 min or treated with higher doses of EGF for 5 min, CRAF interacted with CNK1 and CNK1-bound CRAF was not phosphorylated at its inhibitory phosphorylation site Ser259. AKT and AKT_pT308_ showed only residual binding to CNK1. For each EGF dose tested, 5 min treatment correlated with the strongest ERK phosphorylation. An EGF dose higher than 2 ng combined with stimulation for 15 min or 30 min did not further increase ERK phosphorylation but even diminished ERK phosphorylation. Reduced ERK phosphorylation correlated with elevated binding of active AKT (pAKT_T308_) and inactive CRAF (pCRAF_S259_) to CNK1 complexes. AKT phosphorylation was inversely proportional to ERK phosphorylation. Comparison of the relative amounts of CRAF and pCRAF_S259_ demonstrates that the affinity of CRAF to CNK1 was similar in cells treated with 2 ng and 20 ng EGF. However at 2 ng EGF, CNK1-bound CRAF was not phosphorylated at Ser259 indicating an active CRAF proteins, whereas in cells treated with 20 ng inactive pS259 RAF is bound to CNK1 (Supplementary Figure S5). In case of AKT, a smaller amount of total pT308 AKT bound to CNK1 than AKT not phosphorylated at T308 in cells treated with 20 ng EGF. Thus, subfractions of 2–5% of total cellular RAF and AKT proteins were involved in EGF-induced CNK1 complex formation. Taken together, these data demonstrate that EGF-induced CNK1 complexes alter their composition mediating RAF/ERK activation at low EGF doses and initially at higher doses. By prolonged stimulation with higher EGF doses, AKT suppresses RAF-dependent ERK activation supporting that CNK1 mediates the AKT/RAF crosstalk.

### CNK1 is a differentiation marker promoting skeletal muscle cell differentiation

**Figure 7:**
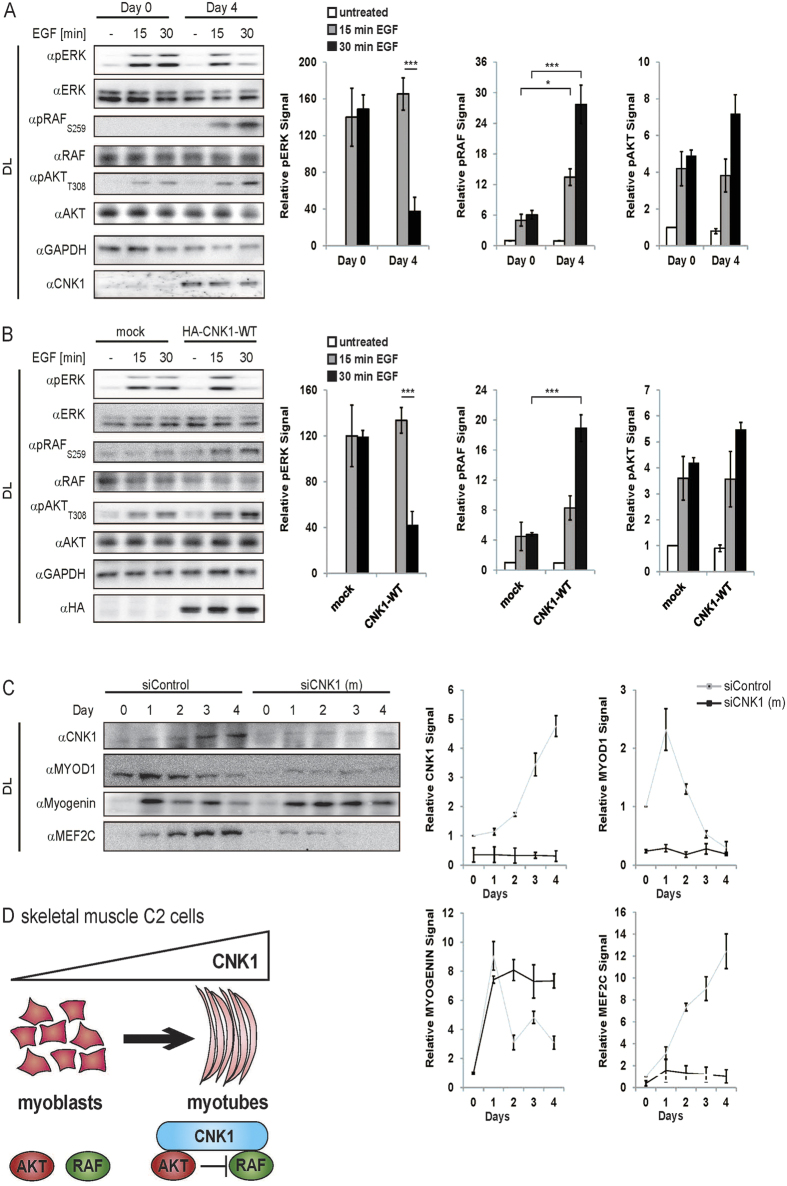
CNK1 is a differentiation marker promoting skeletal muscle cell differentiation. (**A**) EGF (20 ng/ml) stimulates phosphorylation of ERK in proliferating C2 myotubes (day 0). In differentiated C2 cells (day 4) EGF led to transient phosphorylation of ERK since activated AKT inhibits RAF monitored by phosphorylation of Ser259. Bar charts represent quantified data of three independent experiments. N = 3, mean + SEM, two-tailed Students *t-test*, *p < 0.05, ***p < 0.001. (**B**) Expression of CNK1 in C2 myoblasts constitutes the AKT/RAF crosstalk as shown by increased AKT activity, increased inhibitory RAF Ser259 phosphorylation and transient ERK phosphorylation. Bar charts represent quantified data of three independent experiments. N = 3, mean + SEM, two-tailed Students *t-test*, ***p < 0.001. (**C**) Expression of CNK1 is induced in mouse C2 skeletal muscle cells and knockdown of CNK1 interferes with the expression of transcription factors used as differentiation markers. Quantification of CNK1 and transcription factor expression in siRNA and siControl-treated C2 cells is shown. Data obtained from three independent experiments. (**D**) Scheme showing that induction of CNK1 expression enables the AKT/RAF crosstalk controlling differentiation of C2 skeletal muscle cells.

The AKT/RAF crosstalk has been described in different cell types. In most cell types tested including MCF7 cells (see Fig. 4), stimulation of AKT suppresses RAF activity correlating with proliferation of the cells, whereas RAF signalling induces cell differentiation^24^,^30^,^31^. Skeletal muscle cells are an exception^32^. In proliferating myoblasts, AKT and RAF are activated, whereas active AKT inhibits RAF signalling in differentiated multinuclear myotubes. Since CNK1 interacts with AKT and RAF, we analysed whether the expression level of CNK1 may explain this difference. In MCF7 cells, NIH3T3 cells and MEF cells, CNK1 is equally expressed in the proliferative phenotype and in the differentiated or senescent phenotype monitored by the senescence marker p21^CIP^^24^ (Supplementary Figure S6. Contrary, proliferating skeletal muscle C2 myoblasts lacked the expression of CNK1, whereas it was well expressed in differentiated cells (Fig. 7A). Consistent with the low expression of CNK1 in proliferating myoblasts, EGF-stimulated AKT did not phosphorylate Ser259 of CRAF leading to suppression of ERK (Fig. 7A, day 0). On the other hand, in differentiated C2 myotubes, CNK1 expression correlated with an increased level of active AKT, and in consequence thereof with elevated levels of pRAF_S259_ in EGF-treated cells (Fig. 7A, day 4). ERK is stimulated within the first 15 min and then strongly reduced after 30 min when AKT inhibits RAF activity. Ectopic expression of CNK1 in C2 myoblasts restored the AKT/RAF crosstalk, which led to a transient stimulation of ERK similar to differentiated cells expressing endogenous CNK1 (compare Fig. 7B and Fig. 7A, day 4).

Skeletal muscle cell differentiation is controlled by a complex transcriptional network^33^. Differentiation of C2 myoblasts to multinucleated myotubes resulted in decreased MYOD1 expression and increased MEF2C expression, whereas myogenin expression strongly increased with the onset of differentiation and declined during the differentiation process (Fig. 7C). The expression of CNK1 slightly increased during the first two days of differentiation and later showed a strongly increased expression level. Knockdown of CNK1 strongly altered the expression pattern of the differentiation-specific transcription factors (Fig. 7C). Expression of MYOD1 and MEF2C is almost abolished in CNK1 knockdown C2 cells induced to differentiate by serum starvation. In addition, the increased level of myogenin at day 1 of differentiation did not decline during further differentiation but stayed stable. Ectopic expression of CNK1 in C2 cells enhanced the differentiated phenotype and confirms the promoting effect of CNK1 on skeletal muscle cell differentiation (Supplementary Figure S7). In proliferating C2 skeletal muscle cells, the lack of CNK1 expression impaired the execution of the AKT/RAF crosstalk and led to sustained ERK activation. In contrast, expression of CNK1 during differentiation enabled AKT-dependent suppression of RAF and ERK signalling and as a consequence differentiation (Fig. 7D).

## Discussion

Here we identified CNK1 as a molecular platform that controls RAF/ERK and AKT signalling and thereby determines cell fate decisions in a cell type- and cell stage-dependent manner. First, we generated optoCNK1 representing the first optogenetically controlled scaffold protein for dissecting CNK1 signalling. Cluster size of CRY2 fusion proteins differs in respect to the light intensity applied and the time of light exposure^14^,^15^. Accordingly, increased light intensity correlated with increased cluster size of optoCNK1 (Fig. 1B). CNK1 clusters formed at 0.6 μmol m^−2^ s^−1^ contained RAF but no AKT and promoted RAF and ERK activation. Oligomerization of CRAF is a prerequisite for its activation^34^,^35^. CRAF binds with low affinity to CNK1 in non-stimulated cells^6^, and CNK1-bound CRAF may co-oligomerise with light-activated CNK1 leading to CRAF activation in the approach used here. At 2 μmol m^−2^ s^−1^, CNK1 clusters contained RAF and AKT and resulted in inhibition of RAF/ERK signalling. While inhibition of RAF and subsequently ERK can be explained by the AKT/RAF crosstalk, in which AKT phosphorylates and inhibits RAF^24^,^36^, the mechanism of AKT activation is still elusive. For sure, oligomeric CNK1 stimulates AKT as supported by its phosphorylation at Thr308 and Ser473. AKT is phosphorylated by PDK1 at Thr308 and by mTORC2 containing SIN1 as a regulatory compound at Ser473^37^,^38^ indicating that both kinases have to be active under the conditions tested. This resembles constitutively membrane-anchored m/p-AKT and 4-hydroxytamoxifen inducible AKT, both showing phosphorylation of Thr308 and Ser473 and full AKT activity in serum-starved cells^39^,^40^. Interestingly, clusters of CNK1-CRY2 antagonized dark reversion typical for CRY2 oligomers correlating with prolonged AKT activation (Fig. 3). This stabilizing effect can be attributed to the N-terminal SAM domain, a known protein-protein interaction domain^41^. Deletion of the SAM domain prevented binding of AKT to light-induced oligomeric CNK1 and stimulation of AKT by light-activatable CNK1 and, additionally, destabilized CNK1 clusters in darkness. In addition, the SAM deletion in optoCNK1 hampered the AKT/RAF crosstalk and thereby prolonged ERK activation (Fig. 3). Recently we showed that AKT-dependent phosphorylation of the SAM domain induces growth factor-dependent oligomerization and activation of the scaffold CNK1^42^. Thus, the interplay between CNK1 and AKT has different functions. On the one hand, AKT mediates oligomerisation and activation of CNK1 and, on the other hand, CNK1 acts as platform for the AKT/RAF crosstalk.

The function of CNK1 as a molecular platform and switch for RAF/ERK and AKT signalling was further demonstrated in EGF-treated cells to support the physiological relevance (Figs 5 and 6). Increasing concentrations of EGF had similar effects on CNK1 signalling as increased light intensities in case of optoCNK1. At low EGF dose, CRAF bound to CNK1 accompanied with ERK activation. At higher light doses or with prolonged EGF treatment, active AKT was found in the CNK1 complexes together with inactive CRAF correlating with reduced ERK activity. Thus, EGF-stimulated CNK1 and optoCNK1 signal by a similar mechanism.

In EGF-stimulated cells, initial activation of ERK is blocked by the onset of AKT-dependent inhibitory phosphorylation of RAF. Knockdown of CNK1 does not prevent EGF-dependent stimulation of AKT, however, activated AKT no longer inhibits RAF leading to prolonged activation of ERK1. AKT exerts inhibitory phosphorylation of CRAF by Ser259 and of the isoform BRAF by Ser365^36^,^43^. In case of BRAF, AKT and serum and glucocorticoid kinase (SGK) phosphorylate Ser365 inhibiting BRAF kinase activity^36^,^43^. Our data including effects of the AKT inhibitor MK2206 support the notion that AKT inhibits CRAF by phosphorylation at Ser259 in EGF-stimulated cells.

The AKT/RAF crosstalk decides between proliferation and differentiation in several cell types. In MCF7 breast cancer cells, in vascular smooth muscle cells and human embryonic stem cells activated CRAF promotes senescence and differentiation, whereas activated AKT correlates with inhibition of RAF and cell proliferation^29^,^31^,^32^. Implementation of optoCNK1 in MCF7 cells enabled us to induce ERK-dependent differentiation or AKT-dependent proliferation by tuning the light intensity. Thereby, optoCNK1 efficiently mimicked IGF1/AKT-induced proliferation and PMA/ERK-induced differentiation of MCF7 cells^24^ (Fig. 4).

An open question so far was, why in most cell types tested the AKT/RAF crosstalk is exerted in proliferating cells, whereas in mouse C2C12 skeletal muscle cells, this crosstalk occurs at the differentiated stage^32^. We could attribute this cell type- and cell stage-dependent behaviour to the expression level of CNK1. In most cell types CNK1 expression is not altered cell stage specifically (Figure S4). However in C2 cells, CNK1 is not or only slightly expressed in proliferating C2 myoblasts. During the onset of differentiation, expression of CNK1 increased constantly. Ectopic expression of CNK1 in C2 myoblasts constituted a functional AKT/RAF crosstalk and promoted cell differentiation (Fig. 7). Depletion of CNK1 heavily altered the transcriptional network underlying skeletal muscle cell differentiation and impaired the differentiation process. Thus, CNK1 is a target of the transcriptional network that also interferes in transcriptional regulation of skeletal muscle cell differentiation. In a previous study, the 26 kDa phosphatidylethanolamin-binding protein 4 (PEBP4) has been described as a scaffold protein that complexes with CRAF and MEK^44^. PEBP4 enhances differentiation of human skeletal muscle cells and reduced expression of PEBP4 elevates MEK/ERK signalling and inhibits differentiation. PEBP4 interacts with AKT, however, it did not affect the AKT kinase activity and does not regulate AKT in myoblast differentiation^44^. This clearly distinguishes PEBP4 from the multidomain protein CNK1 that forms altering complexes with RAF and AKT and mediates the AKT/RAF crosstalk initiating C2 skeletal muscle differentiation. Several scaffold proteins exist to facilitate RAF/MEK/ERK signalling including kinase suppressor of RAS (KSR), MEK binding partner (MP1) and PEBP4^2^,^45^. However CNK1 serves as a platform for regulation of RAF and AKT signalling.

Taken together, optoCNK1 reveals mechanistic insights underlying the control of RAF and AKT signalling by CNK1. Similar mechanisms we identified in EGF-induced CNK1 signalling indicating that optoCNK1 mimics physiological conditions. We demonstrate that CNK1 mediates the AKT/RAF crosstalk and thereby determines the cell fate as shown here for proliferation *versus* differentiation in a cell type- and cell stage-specific manner. Recently, a functional AKT/RAF crosstalk has been described for a brain ischemia/reperfusion system^46^. During ischemia AKT forms a complex with CRAF, phosphorylates CRAF at its inhibitory site Ser259 and suppresses ERK signalling. During reperfusion reactive oxygen species stimulates the AKT inhibitor PTEN that erases AKT-induced inhibition of RAF/MEK/ERK signalling. Thus, optoCNK1 could be a useful tool to dissect the signal events underlying brain ischemia and reperfusion. In addition, optoCNK1 allows further analysing signalling dynamics and to decipher the function of CNK1 in biological processes. Together with other optogenetic tools, optoCNK1 provides the potential to reprogram a cell in a controlled manner to study processes for tissue engineering and regenerative medicine.

## Methods

### Antibodies and other reagents

CNK1 siRNA were purchased from Qiagen (siCNK1-b, Cat. No. 1027415) and from Santa Cruz Biotechnology (siCNK1-a, sc-142433; siCNK1 (m), sc-142433). Scrambled siRNA used as control was from Santa Cruz Biotechnology (sc-37007). The polyclonal antibodies p44/42 MAPK (ERK1/2), phospho-ERK1/2, CRAF, phospho-CRAF (Ser259), AKT-pan, phospho-AKT (T308), phospho-AKT (Ser473), GSK-3α/β, pGSK-3α/β Ser21/9), SIN, PDK1 and GAPDH antibodies where purchased from Cell Signaling Technology. Antibodies against p21Cip, MEFC2, MYOD1 and Myogenin were from Proteintech. Mouse anti-HA IgG, rabbit anti-HA IgG, anti-mouse IgG-HRP and anti-rabbit IgG-HRP were from Sigma Aldrich, mouse anti-FLAG IgG was from Agilent Technologies, anti-CNK1 was from Santa Cruz. Alexa Fluor^®^ 568 Goat anti-mouse IgG (H+L) and DAPI were from Invitrogen. NILE RED was from Enzo Life Sciences. EGF was purchased from Sigma Aldrich, MK2206, AT7867 and U0126 were from Selleckchem. AKT-kd and AKT-ca where kindly provided by B. Hemmings^47^. MEF2 reporter was purchased from Addgene. CNK1-CRY2 was generated by introducing PCR amplified DNA fragments into the pcDNA3.1-mammalian expression vector (Invitrogen, Carlsbad, CA, USA) using a restriction-enzyme-free isothermal assembly method described elsewhere^48^. All linkers and tags were included into PCR primers (Supplementary Table 1). CNK1 was amplified from the HA-CNK1-WT plasmid. CRY2 was amplified from RAF-CRY2 described in^16^ using primer C_cry2_fw and C_cry2_rv. The corresponding CNK1 fragments were generated using the primer YN_CNK1-fw, bb1_rv, bb1_fw and YN_CNK1-rv. CNK-ΔSAM-CRY2 was generated using primer sets O_AF_33, bb1_rv, bb1_fw and O_AF_37.

### Cell culture, immunoprecipitation and cell staining

HEK293T, HeLa and MCF7 cells were cultured in DMEM supplemented with 10% (v/v) FBS and 1 mM sodium pyruvate. MCF10A cells were cultivated in DMEM-F12 supplemented with 15 mM HEPES, 100 ng/ml cholera toxin, 10 μg/ml insulin, 10 ng/ml EGF, 5% (v/v) horse serum, 100 U penicillin, 100 μg/ml streptomycin, and 0.5 μg hydrocortisone. C2C12 cells were cultivated in DMEM supplemented with 15% (v/v) FBS, 1% NEAA and 2 mM sodium pyruvate. Differentiation medium was DMEM supplemented with 2% (v/v) horse serum and 1% NEAA. NIH3T3 and MEF cells were cultured in DMEM supplemented with 10% (v/v) FBS, 2 mM L-Glutamine, 1% NEAA and 1 mM sodium pyruvate, differentiation was induced by FBS deprivation. Immunostainings was performed as described elsewhere^10^. NILE RED staining was performed according to manufactures protocol. Samples were analysed using a Nikon Eclipse 100 TS microscope, signals were quantified using Image Studio Lite V5.2.

### Transient transfection and cell lysis

Cells were seeded at a density of 70% confluency the day before transfection and starved overnight by using serum-free DMEM (HEK293T and HeLa cells). Plasmids were diluted in Opti-MEM^®^ (Gibco) and PEI solution (1 μg/μl polyethylenimine from Polysciences, pH 7) was added. After 15 min incubation the transfection mix was added to the cells. MCF7 and C2C12 cells were transfected using Lipofectamine 2000 according to manufactures protocol (Invitrogen). 48 h post transfection cells were incubated with lysis buffer (20 mM Tris-HCl pH 7.5, 1% Triton X-100, 100 mM NaCl, 1 mM sodium orthovanandate, 9.5 mM sodium fluoride, 10 mM sodium pyruvate, 10 mM beta-glycerophosphate, and supplemented with protease inhibitors (complete protease inhibitor cocktail, Roche, Basel, CH, cat. No. 04693116001) for 10 min on ice. After suspending, the lysates were boiled in Laemmli sample buffer and separated by 10% SDS-PAGE. Immunoprecipitation was performed overnight by incubation with 1 μl antibody per 400 μl cell lysate on a rotary shaker. After adding 15 μl Sepharose G (Roche) and incubating for further 3 h, samples were washed three times with lysis buffer and resuspended in Laemmli buffer. Immunoblotting was performed using the WET Tank BioRAD-System. Blot quantification was performed using Image Studio Lite V5.2.

### Reporter- and MTT-assays

Reporter assays were conducted as described elsewhere^8^,^10^.

### Optogenetics

Optogenetic experiments were performed described in^16^. In brief, cells were illuminated with self-built boxes containing 460 nm light-emitting diodes with a radiation angle of 120° of which the light intensities were adjusted to 0.6 or 2 μmol m^−2^ s^−1^ using a quantum sensor (LI-250A Light Meter, LI-COR, Lincoln, NE). All experiments took place under red safelight conditions emitted by LEDs (Osram LED DECO^®^ RGB).

### Statistical analyses

All quantitative data are presented as mean +/− SEM, as indicated, of at least three independent experiments by Student’s t Test between group differences. *p < 0.05 **p < 0.01 ***p < 0.001 indicate significance. Immunoblot signals were quantified against the corresponding signal of total antibodies. Microscopy pictures are representative for > 100 cells of at least five independent experiments.

## Additional Information

**How to cite this article**: Fischer, A. *et al*. Optogenetic clustering of CNK1 reveals mechanistic insights in RAF and AKT signalling controlling cell fate decisions. *Sci. Rep.* **6**, 38155; doi: 10.1038/srep38155 (2016).

**Publisher's note:** Springer Nature remains neutral with regard to jurisdictional claims in published maps and institutional affiliations.

## Supplementary Material

Supplementary Information
