# Supplementary material for: Optogenetic clustering of CNK1 reveals mechanistic insights in RAF and AKT signalling controlling cell fate decisions
Source: Sci Rep. 2016 Nov 30;6:38155. doi: 10.1038/srep38155 (PMC5128868; doi:10.1038/srep38155)
Supplement: Supplementary Information [file srep38155-s1.pdf]

Supplemental Information

Optogenetic clustering of CNK1 reveals mechanistic insights in RAF and AKT signaling controlling cell fate decisions

Adrian Fischer, Bettina Warscheid, Wilfried Weber, and Gerald Radziwill

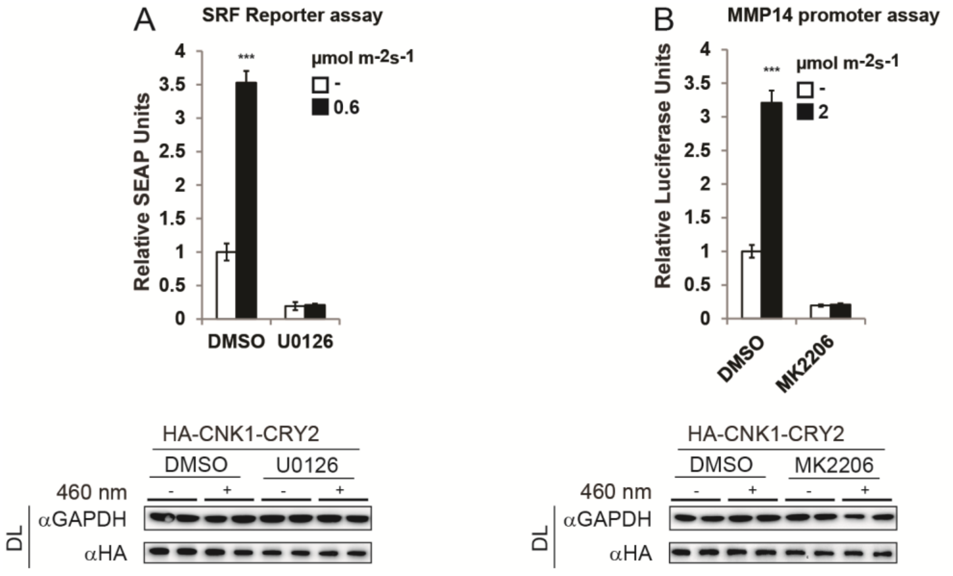

Figure S1. Specificity of reporter assays for ERK and AKT signaling (related to Figure 1 C and D).

(A) SRF reporter stimulation by CNK1-CRY2 exposed to 0.6 ⍰mol $m^{-2}s^{-1}$ depends on MEK/ERK signaling.

(B) MMP14 promoter activation by CNK1 exposed to 2 ⍰mol $m^{-2}s^{-1}$ depends on AKT signaling. N=3, mean + SEM, two-tailed Students *t-test*, ***p < 0.001.

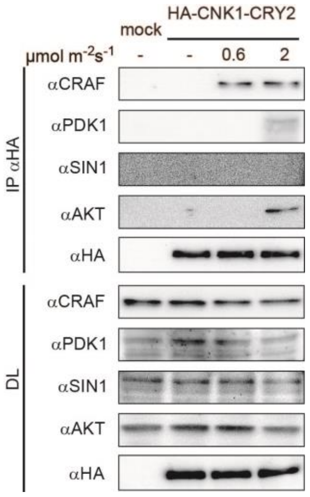

Figure S2. Analysis of CNK1 clusters (related to Figure 1F)

AKT and PDK1 but not SIN1, a key component of mTORC2, co-precipitated with HA-CNK1-CRY2 upon illumination with 2 μmol $m^{-2}s^{-1}$.

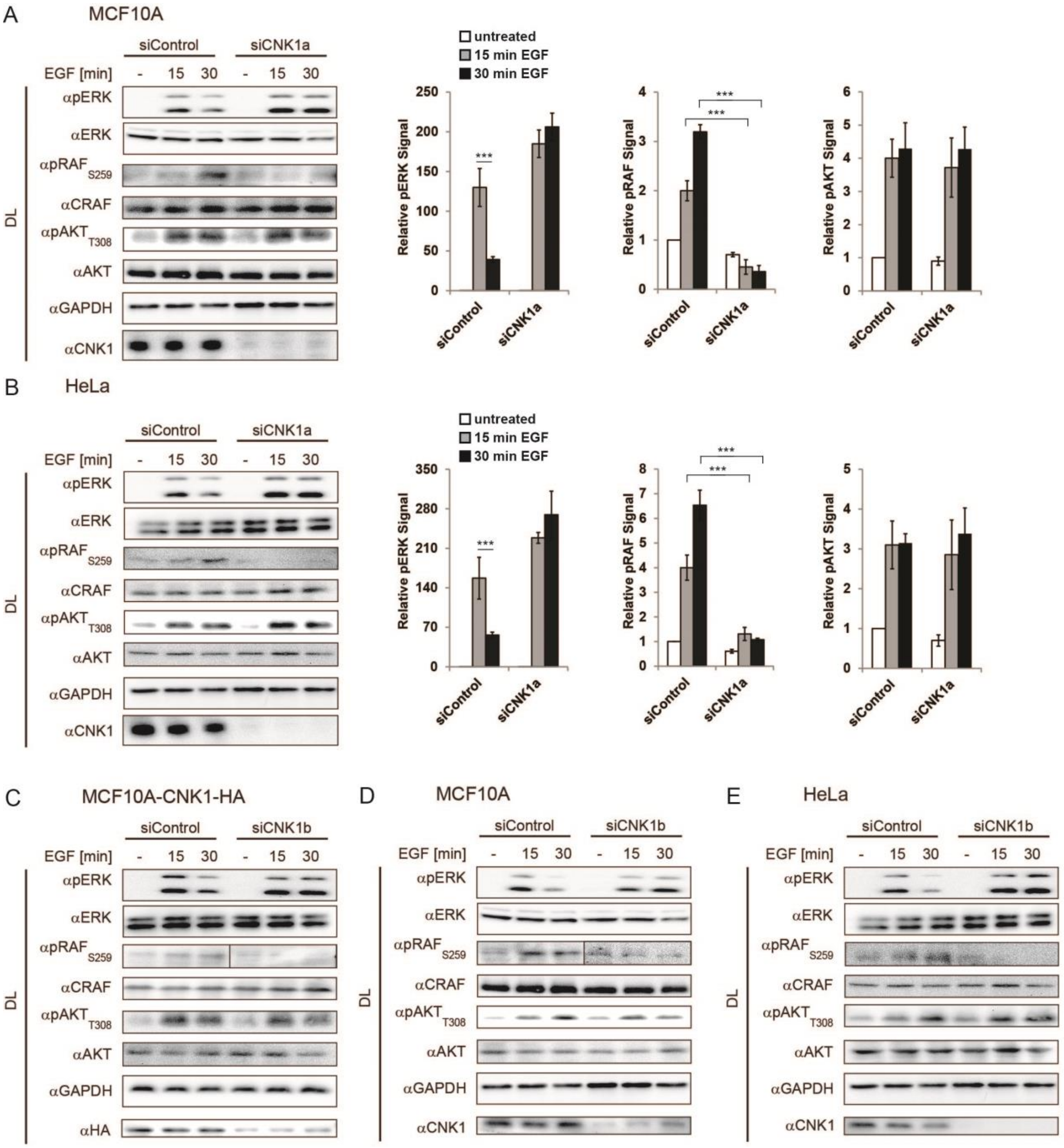

Figure S3. CNK1 mediates AKT-dependent inhibition of CRAF (related to Figure 5).

(A,B) Knockdown of CNK1 by siCNK1a in parenteral MCF10A cells (A) and in HeLa cells (B) abrogates AKT-dependent inhibitory phosphorylation of CRAF at Ser259 leading to increased ERK phosphorylation in EGF (20 ng/ml) stimulated cells. Bar charts represent quantified signals of three independent experiments. N=3, mean + SEM, two-tailed Students *t-test*, ***p < 0.001. **(C, D and E)** Knockdown of CNK1 by siCNK1b confirmed the results obtained by siCNK1a.

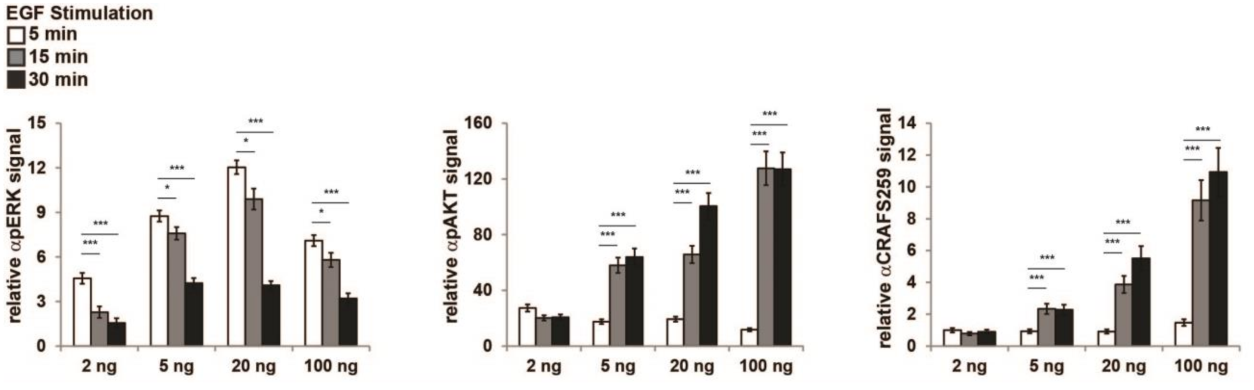

Figure S4. EGF dose-dependent stimulation of ERK, AKT and RAF (related to Figure 6A).

HEK293T cells stimulated with increasing EGF doses for different time periods as indicated. Quantification of the immunoblots shown in Figure 6A, direct lysates.

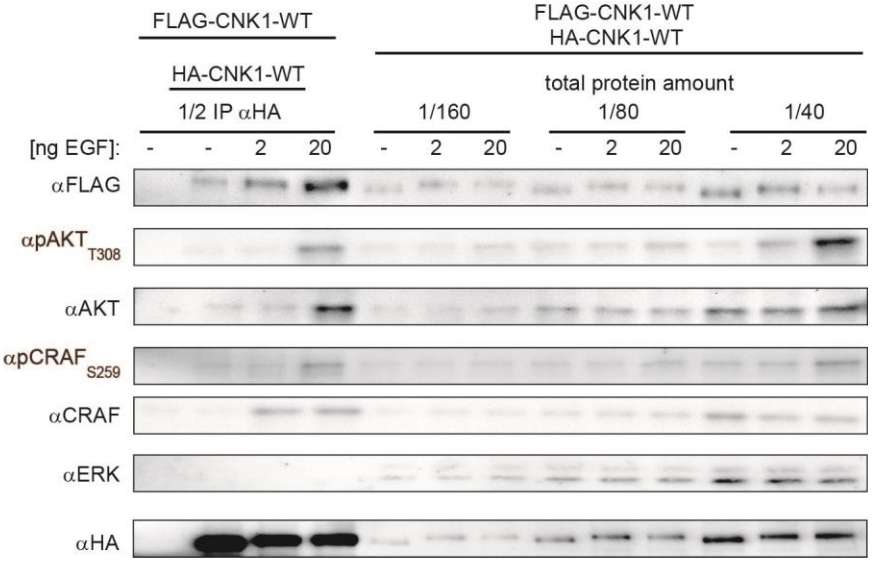

Figure S5. Subfractions of RAF and AKT are complexed with CNK1 in EGF-stimulated cells (related to Figure 6).

HEK293T cells expressing FLAG-CNK1-WT and HA-CNK1-WT were treated with EGF (2ng and 20 ng) for 15 min. Immunopurified HA-CNK1-WT (IP αHA) was analysed for co-precipitated AKT and CRAF proteins. Direct lysates (DL) serve to estimate the fractions of RAF and AKT bound to CNK1.

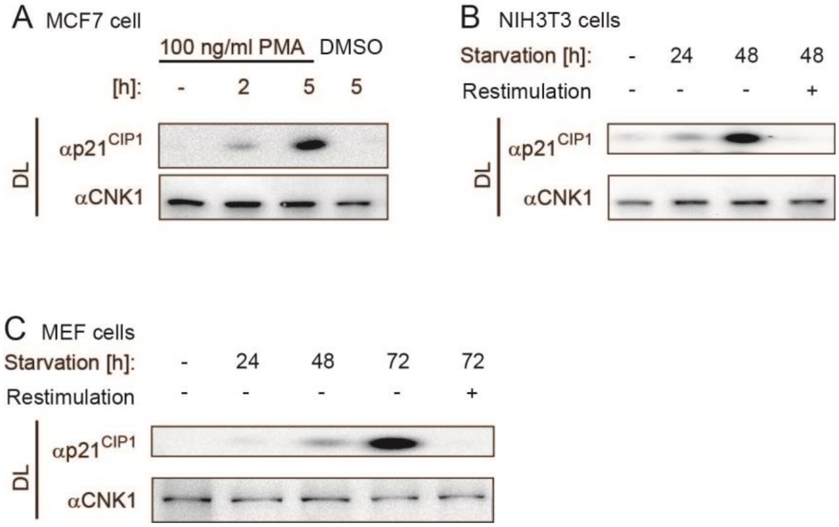

Figure S6. Induction of p21^{CIP} did not affect the CNK1 expression level in MCF7, NIH3T3 and MEF cells (related to Figure 7).

(A) Treatment of serum-starved MCF7 cells with PMA induced expression of p21^{CIP}.

(B) Serum starvation of NIH3T3 cells induced expression of p21CIP reverted by restimulation.

(C) Serum starvation of MEF cells induced expression of p21CIP reverted by restimulation.

(A-C) The expression level of CNK1 was not affected by induction of differentiation.

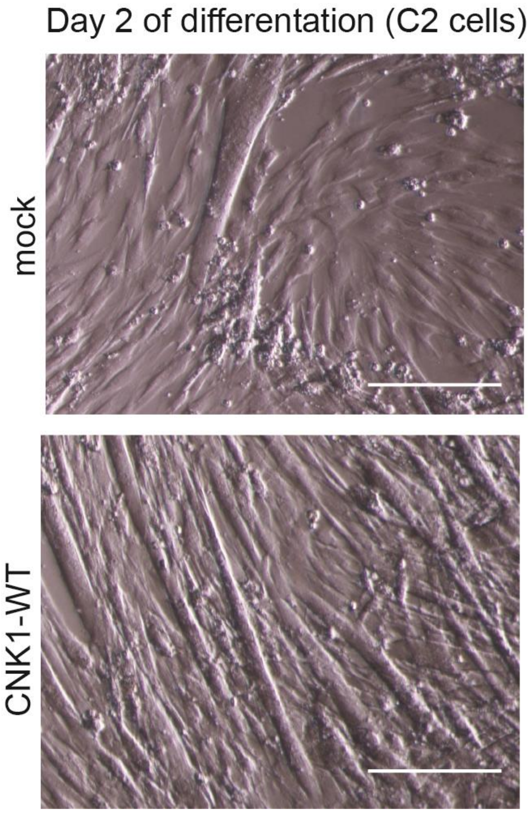

Figure S7. CNK1 accelerates phenotypic differentiation of C2 cells (related to Figure 7).

C2 cells transiently transfected with the CNK1-WT construct showed a more differentiated phenotype compared to mock transfected cells. Scale bar: 100 µm.

Related to Figure 1

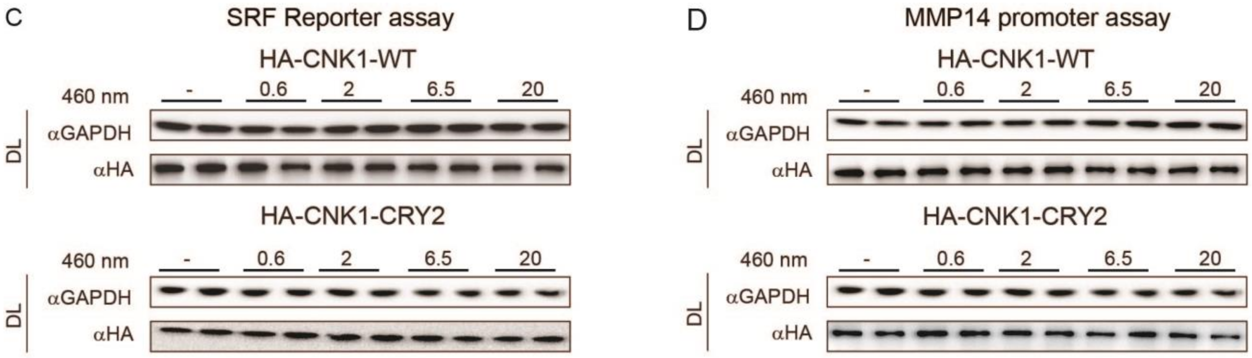

Related to Figure 4

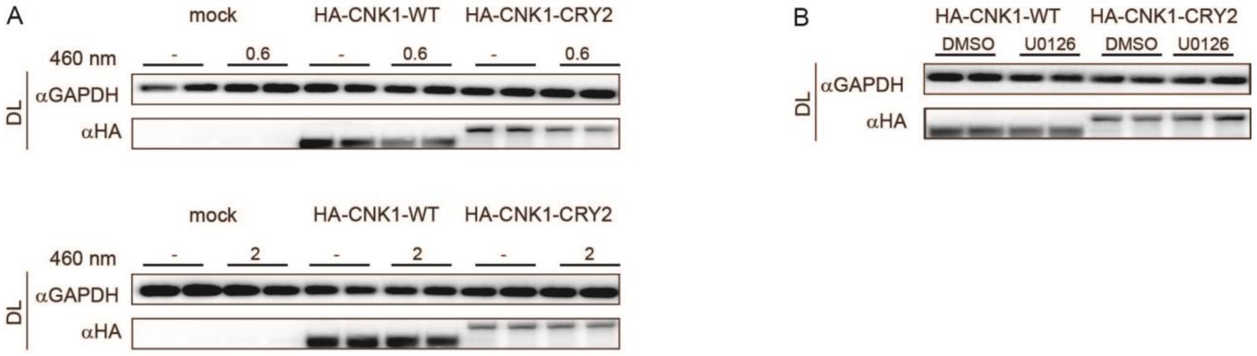

Related to Figure 5

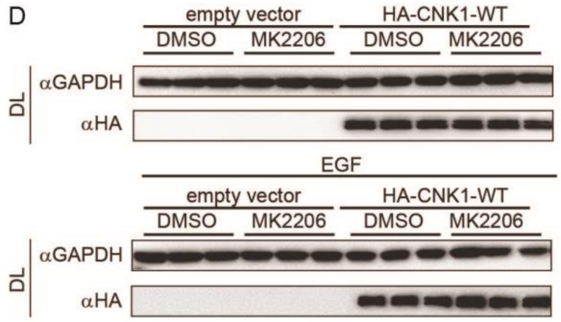

Figure S8. Immunoblots for control of protein expression in reporter assays (related to figures as indicated). Each reporter assay was performed in triplicates. For each reporter assay one set of exemplary blots are shown.

Supplementary table 1: Primer designed and used for PCR amplification in this work.

CNK1-ΔSAM and CNK1-CRY2 Primer	
**Name**	**Sequence**
bb1_forward	CTGCCGCTTACCGGATAC
bb1_reverse	CCACTGAGCGTCAGACC
O_AF_33 forward (delta SAM)	TACGCCTCCCTCATGGAACCGGTAGAGACCAG CTCCAGGCTACAGACAG
O_AF_37 reverse (delta SAM)	CAGGCTTTGCAGGTTCTCTGTCTGTAGCCTGG AGCTGGTCTCTACCGGTTCCATG
YN_CNK1-fw	TACCCATACGATGTTCCAGATTACGCTGAACC GGTAGAGACCTG
YN_CNK1-rev	TCTTTTTGTCCATCTTCATGGTGGCGCGCCGG CGTAGTCGGGCAC
C_cry2_fw	TACCCATACGATGTTCCAGATTACGCTGGCGC GCCACCATG
C_cry2_rev	ACCACCACACCCGCCGCGCTTAATGCGCCGTT ATGCTCCGATCATGATC
